# Diet and condition of mesopredators on coral reefs in relation to shark abundance

**DOI:** 10.1371/journal.pone.0165113

**Published:** 2017-04-19

**Authors:** Shanta C. Barley, Mark G. Meekan, Jessica J. Meeuwig

**Affiliations:** 1 School of Animal Biology and the Oceans Institute, University of Western Australia, Crawley, Western Australia, Perth, Australia; 2 Australian Institute of Marine Science, The Oceans Institute, University of Western Australia, Crawley, Western Australia, Perth, Australia; University of Tasmania, AUSTRALIA

## Abstract

Reef sharks may influence the foraging behaviour of mesopredatory teleosts on coral reefs via both risk effects and competitive exclusion. We used a “natural experiment” to test the hypothesis that the loss of sharks on coral reefs can influence the diet and body condition of mesopredatory fishes by comparing two remote, atoll-like reef systems, the Rowley Shoals and the Scott Reefs, in northwestern Australia. The Rowley Shoals are a marine reserve where sharks are abundant, whereas at the Scott Reefs numbers of sharks have been reduced by centuries of targeted fishing. On reefs where sharks were rare, the gut contents of five species of mesopredatory teleosts largely contained fish while on reefs with abundant sharks, the same mesopredatory species consumed a larger proportion of benthic invertebrates. These measures of diet were correlated with changes in body condition, such that the condition of mesopredatory teleosts was significantly poorer on reefs with higher shark abundance. Condition was defined as body weight, height and width for a given length and also estimated via several indices of condition. Due to the nature of natural experiments, alternative explanations cannot be discounted. However, the results were consistent with the hypothesis that loss of sharks may influence the diet and condition of mesopredators and by association, their fecundity and trophic role. Regardless of the mechanism (risk effects, competitive release, or other), our findings suggest that overfishing of sharks has the potential to trigger trophic cascades on coral reefs and that further declines in shark populations globally should be prevented to protect ecosystem health.

## Introduction

There is strong evidence that apex predators can structure food webs in ecosystems by consuming prey, also known as “lethal effects” [1]. Less well understood are the costly, sub-lethal “risk” effects that predators generate, causing potential prey and competitors to alter their behaviour in order to reduce their risk of being attacked or consumed [2,3]. To avoid predation, prey may shift to safer, less profitable habitats [4,5], forage less actively or efficiently [6,7], alter diet selectivity [8], eat less food or smaller food items [9,10] and become more vigilant [11]. Ultimately, such shifts can impact prey growth rates and reproductive fitness [12,13]. In addition, risk effects can generate niche “partitions” between competitors that reduce conflict over prey items or habitats [12,14]. Removal of a predator can lead to dissolution of niche partitions, allowing competitors to access formerly rare, shared prey and/or expand their diets to include novel prey items [15,16]. Competitive release following loss of a predator may also occur in the absence of risk effects simply due to an increase in the abundance of shared prey items [17].

Sharks are typically considered to be the apex predators on coral reefs [18], yet evidence that they play an important ecological role in these systems is equivocal [19]. Most of the evidence that they impose “top-down” effects on reef fishes has come from relatively small-scale experiments focused not on sharks but on the broader piscivorous guild [20,21], but see [22]. At larger scales (whole reefs), the evidence for or against the importance of sharks comes from large-scale, “natural experiments” where large, carnivorous fishes have been removed by the pervasive and chronic human activity of fishing. Some of these studies have failed to find clear evidence for piscivore-driven trophic cascades on reefs [19,23–30], but a natural experiment conducted at the Northern Line islands, where fishing has created a 5 to 7-fold difference in predator abundance across reefs [29], demonstrated that sites with higher predator abundance were characterised by reductions in several prey attributes including abundance and foraging activity [6], size and longevity [31], condition [32] and size-at-sex change [33]. The role of reef sharks therefore remains ambiguous, with growing evidence that, in certain contexts, they may act as functionally redundant mesopredators that influence reef systems primarily via competition rather than predation [19,34–36].

Comparisons between reefs where fishing has reduced numbers of sharks and reefs where shark assemblages remain intact constitute a natural experiment that can be used to examine the ecological role of these predators [37]. While this approach cannot fully control for alternative factors, it offers one of the few ways to gather evidence at scales relevant to the range of movement of sharks within tropical marine ecosystems (entire reefs and groups of reefs) [38]. However, in most cases, fishing removes not just sharks but also prey and competitor species, such that a reduction in shark-induced effects is compounded by the imposition of a new type of mortality and a loss of select species within the food web [39].

**Fig 1:**
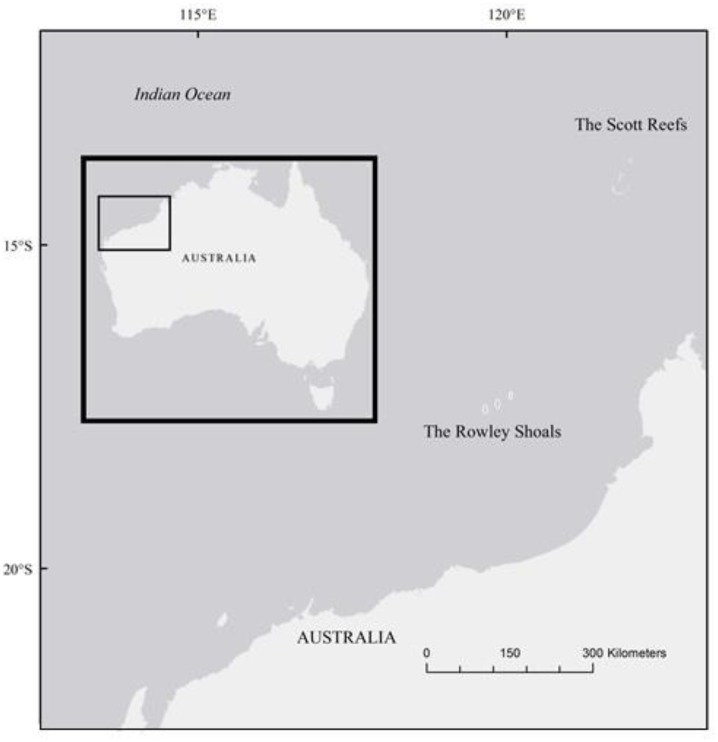
Map of study sites in northwestern Australia. The map depicts the Scott Reefs, where sharks are targeted by Indonesian fishermen, and the Rowley Shoals, where sharks are protected.

Two reef systems located over 300 km from the coast of northwestern Australia offer a unique opportunity to examine how the loss of sharks may influence reef fish assemblages in a situation that is largely free of the secondary effects of fishing [40,41] (Fig 1). The Scott Reefs and the Rowley Shoals have been the subject of the Australian Institute of Marine Science Long Term Monitoring Program for over two decades and are characterised by similar habitat structures, benthic communities, areas (~180 km^2^) and productivity levels, in addition to having well-characterised disturbance histories [42]. Importantly, however, the two locations are sufficiently distant from each other (~400 km) to preclude intermixing of shark and fish populations [43,44]. The Rowley Shoals are a marine protected area that was established in 1990 and has relatively low levels of charter and illegal fishing due to its remoteness from both the Australian mainland and Indonesia [45]. In contrast, sharks have been selectively targeted at the Scott Reefs for centuries by Indonesian fishers, a practice legalised by the Australian government in 1974 [40]. As a result, shark abundance at the Scott Reefs is now 4–17 times lower than at the Rowley Shoals, with some species that are the preferred targets of fishing (e.g. silvertips *Carcharhinus albimarginatus* and scalloped hammerheads *Sphyrna lewini*) almost entirely absent at the former location [46,47]. In contrast, populations of teleosts are still relatively intact at the Scott Reefs as fishers primarily target them for subsistence and do not have the capacity to transport large quantities of teleosts [41]. The contrast in shark abundance between the Scott Reefs and the Rowley Shoals offers the opportunity to examine the importance of these predators within the context of a large-scale (hundreds of km, multiple reefs) natural experiment.

A study by Ruppert *et al*. (2013) found that the shark-depleted fish assemblage at the Scott Reefs had significantly greater numbers of mid-sized carnivores than the Rowley Shoals, suggestive of mesopredator release [42]. The aim of this analysis is to explore whether mesopredators have also undergone changes in diet and condition at the Scott Reefs relative to the Rowley Shoals due to the removal of sharks. In the absence of risk effects, prey have been shown to upregulate activity and preferentially forage in riskier but more profitable habitats relative to prey in predator-rich habitats [20,48,49]. Importantly, risk effects can influence the behaviour of species even when predation rates are low or non-existent [50].

We therefore hypothesized that mesopredators at the Scott Reefs would consume more fish and squid, and fewer benthic invertebrates, than conspecifics at the Rowley Shoals. The pursuit of fish and other prey items typically requires mesopredators to leave the relative safety of the coral reef and venture into the water column, where the risk of attack by predators and/or competitors is higher [20]. In contrast, slow-moving benthic invertebrates can be consumed within the relative safety of the reef and have shorter handling times than fishes [51]. We also hypothesized that loss of competitive effects would lead to a shift in diet from benthic to water column-based prey in mesopredatory teleosts due to breakdown in niche partitioning. Prey differ in calorific content, therefore long-term, consistent prey switching should ultimately result in measurable differences in condition between populations of the same species. We hypothesized that the condition of mesopredators, defined as body weight, height and width for a given length, should improve in response to loss of predation and/or competition due to increased consumption of energy-rich fishes and squids [32]. The evidence for these hypotheses is examined and, within the context of this natural experiment, alternative processes that might also create the observed patterns in diet and condition between these reef systems are considered.

## Methods

This research was conducted under UWA Ethics Approvals: RA3/100/1279, RA3/100/1172 and relevant permits were obtained from the Department of Fisheries (Exemptions 2148 and 2316), Department of Parks and Wildlife (SF009530, SF0010280 and SF009530) and the Department of Sustainability, Environment, Water, Population and Communities (AU-COM2012-170).

### Shark assemblages

Ruppert et al. (2013) estimated that reef sharks (primarily silvertip *Carcharhinus albimarginatus* and grey reef *Carcharhinus amblyrhynchos*) were ~3 times more abundant at the Rowley Shoals than at the Scott Reefs, based on baited remote underwater video systems (BRUVS). The mean of the maximum number of sharks seen together in a single frame of BRUVS footage was 0.4 per hour at the Scott Reefs vs ~1 per hour at the Rowley Shoals [42]. Whereas the Scott Reefs shark assemblage is characterised primarily by relatively small, “mesopredatory” shark species such as grey reef *C*. *amblyrhynchos*, zebra *Stegostoma fasciatum* and white tip reef *Triaenodon obesus*, the Rowley Shoals assemblage additionally contains larger, species such as silvertip *C*. *albimarginatus*, tiger *Galeocerda cuvier*, scalloped hammerhead *Sphyrna lewini* and great hammerhead *Sphyrna mokarran* [47].

### Abundance data

Data on fish abundance for the five focal species (see below) and an additional eleven other mesopredators (**Table A in** S1 File) were extracted from the Long Term Monitoring Program (LTMP) database of the Australian Institute of Marine Science (AIMS), which annually surveyed three sites per location (the Scott Reefs and the Rowley Shoals) between 1994 and 2008. Each site was comprised of five, 50 m-long transects marked with star pickets every 10 m at a depth of 6–9 m. Transects were 10 m apart. For each survey, a trained scuba diver counted large, mobile species at a distance of up to 2.5 m on either side of the transect line while swimming at a speed of 10 m/min. An assistant swam 10 m behind the surveyor, laying a tape measure on the sea floor. Once the five transects were complete, the surveyor returned along the tape measure and counted small, site-attached species.

### Fish collections

Fishes of five focal species were collected at the Scott Reefs in November 2012 and February 2014 and at the Rowley Shoals in April 2013 and November 2013. Up to 90 individuals of *Lutjanus gibbus*, *Lutjanus decussatus* and *Lutjanus bohar* and 50 individuals of *Lutjanus kasmira* and the lethrinid *Monotaxis grandoculis* were sampled by free divers using spear guns at each location (Table 1). Fishes were collected at depths of up to 20 m, euthanised using the *iki jime* method and stored in an ice slurry for a maximum of three hours. Body dimensions in mm (standard, fork and total length) were measured using a purpose-built fish board with inset tape measure. Digital calipers were used to measure body height at the anterior edge of the dorsal fin and body width at the first ray of the dorsal fin, while total wet weight (g) was recorded using a digital balance to an accuracy of 0.01 g. Sex and sexual stage based on [52] were also recorded following examination of the gonads. Guts were removed and preserved in 10% formalin for a month, before being washed in water and the formalin replaced with 70% ethanol.

**Table 1:** Sample size (n) with % females in parentheses. Also shown are mean (range) values for length of five mesopredatory fishes at the Scott Reefs and the Rowley Shoals. The p-values for results of a 2 x 2 contingency table with no fixed margins (% females) and a two-sample t-test assuming unequal (*L*. *decussatus*, *L*. *kasmira* and *L*. *bohar*) and equal (*L*. *gibbus*, *M*. *grandoculis*) variances (total length) are shown.

	n (% females)			Total length (cm)		
Location	Scott	Rowleys	*p*	Scott	Rowleys	*p*
*L*. *gibbus*	87 (32.1)	87 (39.0)	ns	330.8 (260–399)	343.4 (266–402)	0.005
*L*. *decussatus*	87 (63.3)	78 (52.3)	ns	223.8 (195–258)	239.0 (199–262)	3.7E-05
*L*. *kasmira*	49 (56.0)	43 (57.8)	ns	211.3 (187–237)	223.2 (192–241)	0.0001
*M*. *grandoculis*	50 (38.1)	50 (53.1)	ns	279.2 (199–462)	288.1 (204–483)	ns
*L*. *bohar*	86 (38.5)	90 (55.1)	0.025<*p*<0.05	484.1 (221–715)	515.4 (230–713)	ns

We chose to study *L*. *gibbus*, *L*. *decussatus*, *L*. *kasmira*, *M*. *grandoculis* and *L*. *bohar* because they consume a range of benthic invertebrates and teleosts and therefore have the potential to display dietary shifts in response to changes in risk effects. In addition, the five species occupy relatively high trophic positions within reef food webs and therefore have the potential to act as both the prey and competitors of reef sharks. Moreover, the species differ in life history (e.g. maximum size, size at reproduction, longevity, foraging behaviour) allowing for the generality of the findings to be examined. For example, *L*. *gibbus* reaches a maximum length of 50 cm, while *L*. *decussatus*, *L*. *kasmira*, *M*. *grandoculis* and *L*. *bohar* attain lengths of 35 cm, 40 cm, 60 cm and 90 cm, respectively [53]. Lutjanids and lethrinids consume crabs, bivalves, gastropods, echinoderms, worms, cephalopods, shrimp and small teleosts [54]. Although the diet of sharks is poorly understood, there is evidence that reef-associated sharks consume both lutjanids and a diverse array of herbivorous fishes including acanthurids and scarids [34,55–57]. In addition, both reef sharks and lutjanids, including *L*. *bohar*, *L*. *gibbus* and *L*. *decussatus*, are primarily nocturnal feeders, although evidence is limited [58,59]. As a result, the latter are likely to experience stronger risk effects than diurnally active fishes, although risk effects can occur without direct consumption [50,60,61].

### Data analysis

#### Comparability of collections and locations

The LTMP dataset was filtered for the 16 most abundant mesopredators and years common to both locations. Data from 1994, 1999, 2003 and 2004 were excluded because only one location was sampled in these years. Mean abundances per site and location (the Scott Reefs and the Rowley Shoals) were calculated for each species (**Table A in** S1 File). Mean abundance was compared between locations using a paired two sample for means t-test. Mean abundance data for each species in each location was then analysed using least squares regression. We used a 2 x 2 Chi-Square contingency table with no fixed margin to determine whether sex ratios of sampled fishes differed between locations. Total lengths of each species were compared between locations using a t-test based on the log10 transform of individual values. Data series of temperature for the same time period and depths were available for single sites on the reef slopes of the Scott Reefs (6 m) and the Rowley Shoals (Mermaid Reef, 7 m) between June 2007 and August 2012 from the AIMS LTMP temperature database [62]. Mean monthly average temperature at each location was compared using a paired t-test.

#### Gut content analysis

The volume and wet mass (formalin-fixed and ethanol-preserved) of intact guts from *L*. *gibbus*, *L*. *decussatus* and *L*. *bohar* were measured and prey items extracted. The presence of scales, jaws and other items were used to identify fishes, while eyes, carapaces and chelipeds were used to identify crustaceans. The length and morphology of beaks were used to distinguish between squid and octopi [63]. Prey items were identified to species where possible using compound and dissection microscopes and photographed. Individuals that had empty stomachs were excluded from the analysis. Prey items were pooled into ten categories (Table 2) and two broader groups: “water column” and “benthic”. The percentage of water column-derived prey items found in the guts was calculated for each individual to account for the fact that some mesopredators contained more than one prey item. To assess whether the percentage of prey items in the guts that originated in the water column was significantly higher at the Scott Reefs than at the Rowley Shoals, a 2×2 Chi-Square contingency table with no fixed margin was used.

**Table 2:** Number of prey items and their percentage contribution (in parentheses) in the guts of *L*. *gibbus*, *L*. *decussatus* and *L*. *bohar* at the Scott Reefs and the Rowley Shoals. Sample sizes for each species and reef system are shown in square brackets.

	*L*. *gibbus*		*L*. *decussatus*		*L*. *bohar*	
	Scott [n = 35]	Rowleys [n = 40]	Scott [n = 37]	Rowleys [n = 28]	Scott [n = 37]	Rowleys [n = 34]
**Benthic**						
Bivalve	6 (8.8)	13 (13.7)	0 (0.0)	0 (0.0)	1 (2.0)	1 (1.0)
Crab	21 (30.9)	31 (32.6)	11 (22)	20 (50)	8 (16)	58 (59.2)
Echinoderm	0 (0.0)	2 (2.1)	0 (0.0)	0 (0.0)	0 (0.0)	0 (0.0)
Gastropod	12 (17.6)	32 (33.7)	0 (0.0)	1 (2.5)	0 (0.0)	1 (1.02)
Isopod	0 (0.0)	0 (0.0)	0 (0.0)	1 (2.5)	0 (0.0)	1 (1.02)
Marine worm	2 (2.9)	1 (1.1)	0 (0.0)	1 (2.5)	0 (0.0)	2 (2.04)
Octopus	0 (0.0)	0 (0.0)	0 (0.0)	0 (0.0)	0 (0.0)	2 (2.04)
**Water Column**						
Fish	21 (30.9)	12 (12.6)	27 (54)	13 (32.5)	33 (66)	30 (30.6)
Shrimp	4 (5.9)	0 (0.0)	10 (20)	2 (5.0)	6 (12)	0 (0.0)
Squid	2 (2.9)	4 (4.2)	2 (4.0)	2 (5.0)	2 (4.0)	3 (3.06)

#### Condition

We calculated length–weight relationships, where condition was estimated from the residuals of an ordinary least squares regression of log-transformed body mass against length, following [64]. Lengths (L; mm) and weights (W; g) for each fish were first log10 transformed to stabilize variance. A dummy variable (DV = 1 for the Scott Reefs, DV = 0 for the Rowley Shoals) was included to test for a main effect (DV) and the presence of an interaction *DV* × log *L*. The general model was log *W* = *b*_0_ + *b*_1_ × log *L* + [*b*_2_ × *DV*] + [*b*_3_ × log *L* × *DV*]. Predicted weight at the minimum, median and maximum lengths was back-calculated (10^log *W*^) for each species at each location and the difference between the two estimates calculated as the percentage difference in weight:

$$\%\Delta W=100\times \left(\frac{\left[W_{SCOTT}-W_{ROWLEYS}\right]}{W_{ROWLEYS}}\right)$$

This approach was repeated for body height (%ΔH) and width (%ΔWi) on length using non-transformed data. Two independent indices of condition, including Fulton’s condition factor (K) and relative condition factor K_n_ were also calculated (**Methodology in** S1 File).

## Results

### Comparability of collections and locations

**Fig 2:**
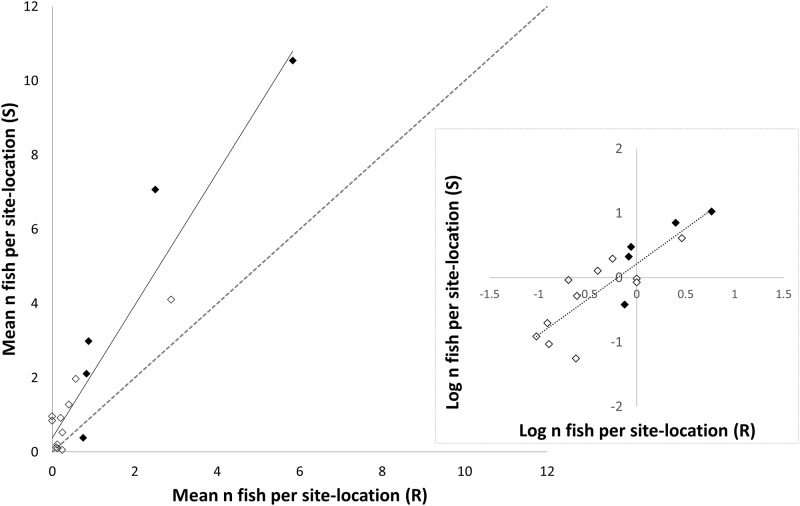
Plot of regression of mean number (n) of fish per site-location (abundance, A) at the Rowley Shoals (R) against the Scott Reefs (S) for 16 species of mesopredatory teleost where A_S_ = 1.79×A_R_+0.38 (n = 16, *p* = 1.7×10–8, R^2^ = 0.90, α = 0.05). Abundance data were sourced from the Australian Institute of Marine Science Long-Term Monitoring Program. The plot includes the five study species (black) and eleven non-focal species (white). The dashed line shows a 1:1 relationship between abundances at each location. Abundance was averaged across 3 sites at each location (Clerke, Imperieuse and Mermaid at the Rowley Shoals and North Scott, South Scott and Seringapatam at the Scott Reefs). The inset figure shows logged values for mean fish abundance per site-location. See **Table A in** S1 File and the AIMS website for details:http://www.aims.gov.au/docs/research/monitoring/monitoring.htm.

The fish assemblage at the Scott Reefs was characterized by greater numbers of mesopredators than the Rowley Shoals (paired two-sample for means, one-tailed t-test: *p =* 0.004, *t*_16_ = 1.75). The slope of the regression of mesopredator abundance at the Scott Reefs vs. abundance at the Rowley Shoals was significantly different from 1 (b_1_ = 1.79 ± 0.16; Fig 2). At the Scott Reefs, *L*. *gibbus*, *L*. *decussatus*, *L*. *kasmira* and *M*. *grandoculis* were 2.8, 1.7, 1.9 and 1.6 times more abundant than at the Rowley Shoals, respectively. In contrast, *L*. *bohar* was 0.5 times less abundant at the Scott Reefs than at the Rowley Shoals.

A total of 707 fish of the five focal species were collected from the Scott Reefs and the Rowley Shoals (Table 1). The sex of 70% of these fishes was determined, with sex ratios consistent between locations for all species except *L*. *bohar* (2×2 contingency table with no fixed margins: χ^2^ = 4.9, 0.025<*p*<0.05). Mean length was determined to be similar at the Scott Reefs and the Rowley Shoals for *M*. *grandoculis* (two-sample, two-tailed t-test assuming equal variances: *p =* 0.5, *t*_100_ = 0.68) and *L*. *bohar* (*p =* 0.05, *t*_176_ = 2.0), but differed for *L*. *gibbus* (*p =* 0.005, *t*_174_ = 2.8), *L*. *decussatus* (*p =* 3.7E-05, *t*_165_ = 4.24), and *L*. *kasmira* (*p =* 0.0001, *t*_95_ = 4.0; **Fig A in** S1 File). There was no significant difference in paired monthly temperatures between the two locations (paired two-sample, two-tailed t-test for means: *p =* 0.45, *t*_82_ = 0.76).

### Gut content analysis

**Fig 3:**
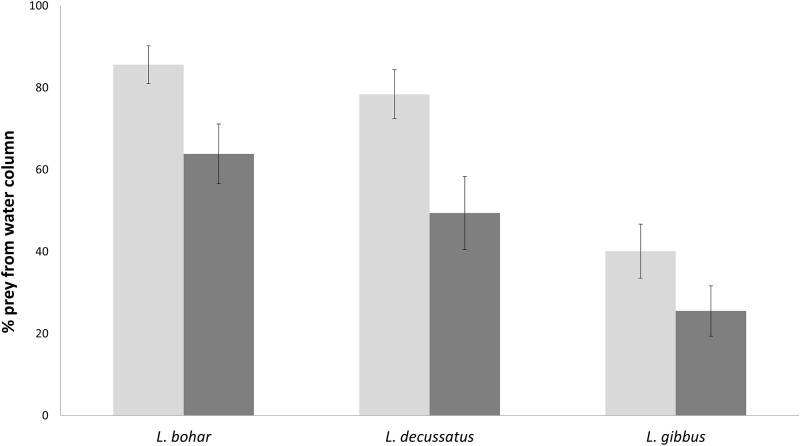
Prey items in guts of three mesopredatory teleost species by origin. Plot of the mean percentage of prey items from the water column (±SE) in the guts at the Scott Reefs (pale grey) and the Rowley Shoals (dark grey) for three species of mesopredator (2x2 Chi-Square contingency table with no fixed margin: *L*. *bohar*, χ^2^ = 30.9, *p*<0.001, *L*. *decussatus*, χ^2^ = 11.9, *p*<0.001 and *L*. *gibbus*, χ^2^ = 10.7, 0.001<*p*<0.005).

A total of 401 prey items were extracted from the stomachs of 75 *L*. *gibbus*, 71 *L*. *bohar* and 65 *L*. *decussatus* (Table 2). Guts were dominated by fish and crabs, although other benthic prey items such as gastropods were also abundant, particularly in *L*. *gibbus*. For all mesopredators, counts of water column-derived prey items were significantly higher at the Scott Reefs than at the Rowley Shoals (2×2 contingency table with no fixed margins; *L*. *gibbus*, 40.1±6.6% vs 25.5±6.1%, χ^2^ = 10.7, 0.001<*p*<0.005; *L*. *decussatus*, 78.4±6.0% vs 49.4±8.9%, χ^2^ = 11.9, *p*<0.001; *L*. *bohar*, 85.6±4.6% vs 63.8±7.3%, χ^2^ = 30.9, *p*<0.001; Fig 3). Among the prey found in the guts of *L*. *bohar* were fishes belonging to Scaridae, Sygnathidae (*Corythoichthys* spp), Lethrinidae (*M*. *grandoculis*), Serranidae (possibly *Epinephelus fasciatus*), Acanthuridae, Balistidae and Chaetodontidae. Scarids were also found in the guts of *L*. *decussatus* while echinoderms were only found in the guts of *L*. *gibbus* at the Rowley Shoals.

### Condition

**Fig 4:**
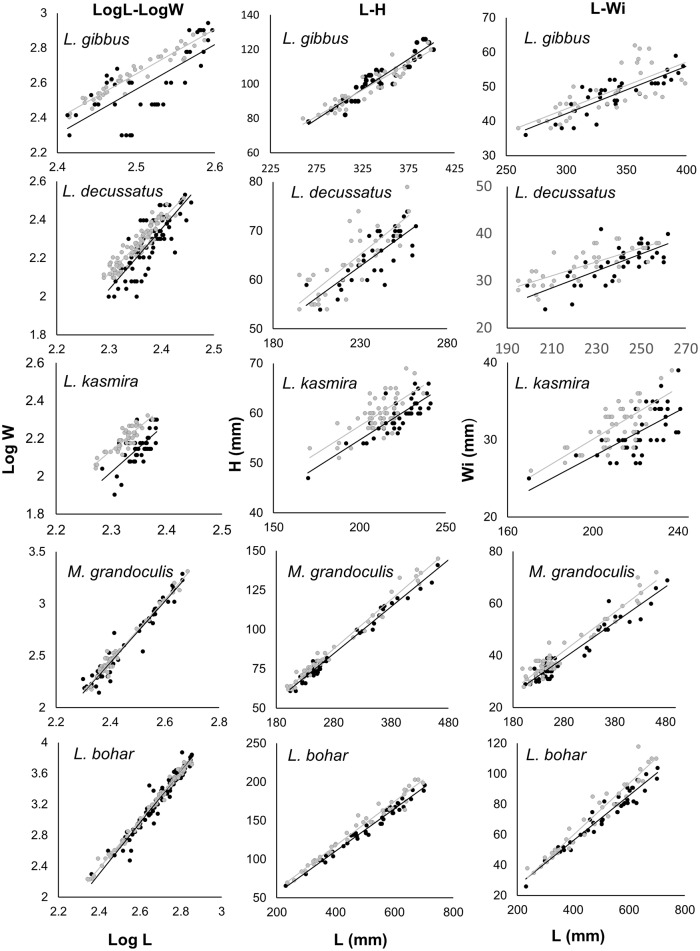
Regressions of Log length (Log L) on Log weight (Log W), Length (L) on height (H) and Length (L) on width (Wi) for *L*. *gibbus*, *L*. *decussatus*, *L*. *kasmira*, *M*. *grandoculis* and *L*. *bohar* at the Scott Reefs (grey) and the Rowley Shoals (black). Differences in the regression lines between species might be obscured due to variation in the range of the axes. See **Table C in** S1 File for statistics.

All linear regressions on log-transformed length and weight data were significant with a clear effect of location for *L*. *bohar*, *L*. *decussatus* and *L*. *kasmira* (Fig 4; **Table C in** S1 File). All species were heavier for a given length at the Scott Reefs relative to the Rowley Shoals including *L*. *gibbus* (+7.6%), *L*. *decussatus* (+14.6%), *L*. *kasmira* (+28.2%), *M*. *grandoculis* (+7.5%) and *L*. *bohar* (+8.8%; **Table B in** S1 File). *Lutjanus bohar* was the only species that had a significant interaction between length and location, with an inflection point at 601 mm. Below this length, *L*. *bohar* were heavier for a given length at the Scott Reefs than at the Rowley Shoals. At the observed median length of 468 mm, fish were 8.8% heavier at the Scott Reefs than the Rowley Shoals, while at the maximum length of 715 mm, animals at the Scott Reefs were 2.4% lighter (see **Fig B in** S1 File).

Linear regressions on height-length data were significant for all species, with an effect of location for all species except *L*. *gibbus* and no interactions (**Table C in** S1 File). Respectively, *L*. *decussatus*, *L*. *kasmira*, *M*. *grandoculis* and *L*. *bohar* were 4.1%, 5.1%, 3.5% and 8.6% deeper bodied for a given length at the Scott Reefs than at the Rowley Shoals (**Table B in** S1 File).

Linear regressions on width-length data were significant for all species except *L*. *gibbus*, while *L*. *bohar* had a significant interaction between location and the width-length relationship. *Lutjanus decussatus*, *L*. *kasmira* and *M*. *grandoculis* were 5.9%, 9.2% and 7% wider, respectively, for a given length at the Scott Reefs than the Rowley Shoals. The significant interaction between length and location for *L*. *bohar* (linear regression, DVxL, *p =* 0.021, n = 78) meant that while individuals were 5.1% wider at the median length at the Scott Reefs than at the Rowley Shoals, the smallest animals were no different in width between the two locations (*L*_*min*_: 0.2%) yet substantially wider at the Scott Reefs at the largest sizes (10.3% wider at 702 mm).

## Discussion

Our results are consistent with the hypothesis that reef sharks influence the diet and condition of mesopredators on coral reefs. On shark-depleted reefs, mesopredatory fishes were relatively abundant and fed on a diet that was largely composed of fishes and squid, whereas at the Rowley Shoals, where higher densities of sharks were present, abundances of mesopredators were lower and the diets of the same species were dominated by benthic invertebrates. These differences in diet were accompanied by variation in morphology and condition, such that fishes at the Scott Reefs tended to be heavier, deeper-bodied and wider for a given body length than fishes of the same species at the Rowley Shoals. The results of this study suggest that sharks may impose significant top-down effects on reef fish assemblages. However, it is unclear whether the mechanism by which these predators structure ecosystems is based on risk effects, competition, or a mixture of both.

Consistent with the hypothesis that reef sharks are important predators that impose risk effects on potential prey, mesopredators consumed a greater proportion of low-risk, benthic food items at the Rowley Shoals relative to conspecifics at the Scott Reefs. As a prey source in a predator-rich environment, benthic invertebrates living within the interstices of the reef represent a less risky prey option than fishes or squid, which are typically located in the water column, where risk of predation is higher [65]. Fishes are also faster-moving, rove over larger areas, require more activity and energy to capture and have longer handling times than benthic invertebrates [66,67]. Additionally, the act of predation on reef fishes generates alarm cues, alerting other prey to the presence of the predator and initiating learning about predator behaviours that makes future prey harder to catch [68,69]. Together, these factors make fish and squid far riskier prey items than benthic invertebrates for mesopredators in situations where they, in turn, are subject to the threat of being consumed. Our results are consistent with previous studies showing that dugongs, bottlenose dolphins and turtles manage their risk of being attacked by tiger sharks by foregoing energetically profitable food in the centre of sea grass meadows, where escape options are limited, in favour of lower quality food on the edge of meadows [4,5,70].

It is entirely possible that the shift in diet recorded in mesopredators at the Scott Reefs relative to the Rowley Shoals reflects competitive rather than predatory release. There is growing evidence that sharks and large piscivores such as lutjanids and serranids share food resources and occupy overlapping trophic niches [19,34]. A reduction in the number of sharks at the Scott Reefs may not only have increased the availability of formerly rare, shared prey species to mesopredators, but also weakened niche partitions, allowing mesopredators to consume novel teleost prey that were previously off-limits. This would be consistent with previous studies in a range of taxa that have demonstrated niche expansion following the loss of a competitor [17,71,72]. Declines in the blue shark *Prionace glauca*, for example, led to changes in habitat use by the salmon shark *Lamna ditropis*, potentially causing the latter to incorporate new cephalopod prey species into their diets [73]. The finding that competition may be an important mechanism by which sharks structure reef fish assemblages would be consistent with recent studies that have concluded that reef sharks may operate as functionally redundant mesopredators rather than apex predators [19]. However, it is likely that sharks regulate fish assemblages via a complex mixture of both competitive and predatory effects that is continuously evolving to reflect the relative sizes of the reef sharks and teleosts within the population.

A diet of fish (5.4–6.1 kcal/g) and squid (5.5 kcal/g) is far more energetically rewarding than one composed of relatively low energy prey such as crustaceans (3.4–3.7 kcal/g) and gastropods (1.1 kcal/g), in addition to being less energetically costly to digest and quicker to assimilate [74]. Diet is one of the key determinants of condition [75]. To test if the observed shift in mesopredatory diet from invertebrates towards higher-quality prey from the water column was correlated with improved condition, we compared the body dimensions of mesopredators at the Scott Reefs and the Rowley Shoals. All five mesopredators were significantly heavier for a given length at the Scott Reefs compared to the Rowley Shoals. The regression of log-transformed body mass against length for *L*. *bohar* was characterized by an inflection point at 61 cm, above which there was effectively no difference in condition between individuals at Scott Reefs and the Rowley Shoals. Given a mean maximum length of the two dominant species of reef shark at the two locations of 1520 mm as measured on stereo-Baited Remote Underwater Video Systems [76], *L*. *bohar* may enter into a size refuge at a length of ~55 cm as reef sharks typically do not consume prey >36% of their length [77]. Moreover, predators tend to feed on prey between one and three orders of magnitude smaller in mass than themselves [78]. Given a maximum published mass for *C*. *amblyrhynchos* of ~34 kg, prey over 3.4 kg are therefore unlikely to be consumed (but see [79]). Length-weight regressions suggest that an *L*. *bohar* of this weight is ~59 cm, corroborating size refugia as a possible explanation for the inflection point. Footage from the stereo-BRUVS also support the premise that large *L*. *bohar* escape predation at larger sizes, as they depict large individuals feeding aggressively on bait in the presence of sharks [76].

In addition to being heavier for a given length at the Scott Reefs relative to the Rowley Shoals, mesopredatory fishes at the former location were also significantly deeper bodied for a given length at the shark-depleted location. This finding is consistent with studies of body shape in the Bahamas mosquitofish *Gambusia hubbsi*, which found that low-predation environments selected for fishes with deeper bodied anterior regions than high-predation environments, potentially due to a reduced need to engage in fast-starts [80]. Larger body depth may also have been selected for at the Scott Reefs due to increased competition between mesopredatory fishes following a reduction in shark numbers. Fishes with greater body depths can swim for more prolonged periods of time and may therefore be better able to secure prey, access mates and conserve energy [80]. The lack of evidence for a significant increase in height for *L*. *gibbus* at the Scott Reefs compared to the Rowley Shoals was surprising, however *L*. *gibbus* may be anatomically constrained in body height, as it is deeper-bodied than other lutjanids [81].

Mesopredatory fishes were also significantly wider for a given length at the Scott Reefs than at the Rowley Shoals. *Lutjanus decussatus* were 5.9% wider at the median length at the Scott Reefs than at the Rowley Shoals, while *L*. *kasmira*, *M*. *grandoculis* and *L*. *bohar* were 9.2%, 7.0% and 5.1% wider, respectively. Increased width acts as a good measure of condition because it is indicative of better developed fat reserves and propulsive muscles in the caudal region [82]. Indices such as Fulton’s K factor also confirmed that all mesopredatory fishes were in significantly better condition at the Scott Reefs compared to the Rowley Shoals. Improved condition among mesopredatory fishes at the Scott Reefs may be a consequence of the observed shift in their diet towards fish prey relative to conspecifics at the Rowley Shoals. However, a reduction in the abundance of sharks at the Scott Reefs may also have promoted the condition of mesopredatory fishes by allowing them to forage more effectively and for longer periods [7,83,84], waste less energy on costly escapes from attacks [49,85,86] and experience fewer physiological stress responses, which can trigger muscular protein catabolism via increased plasma corticosterone levels [87–89].

Paired reef systems constitute a useful window into the debate on the role of sharks on coral reefs. However our results must be interpreted cautiously in light of the fact that natural experiments are not replicated, randomized and controlled trials [37]. Indeed, one possible alternative explanation for the observed results is that the temperature regime at the two locations differed. However, we could find no consistent differences in average water temperatures between locations. In addition, it is possible that the results were influenced by other differences between the locations and/or the sampling design. However, Ruppert et al. (2013) argued that the two reef systems were similar in terms of productivity, habitat structure, the composition and average cover of reef size, benthic communities and the frequency, intensity and history of disturbance. Moreover, it is impossible to exclude the possibility that the results were an artefact of intra- and inter-annual variation. As it was logistically unfeasible to collect all samples at the same time at both locations, fishes were collected at the Scott Reefs in February 2014 and November 2012 and at the Rowley Shoals in April 2013 and November 2013. Temporal fluctuations in environmental conditions may therefore have influenced the results.

It is also possible that the recorded differences in the diet and condition of mesopredatory fishes between locations reflected differences in the relative availability of their prey rather than predation risk. However, herbivores and other potential prey of mesopredatory fishes were significantly more abundant at the Rowley Shoals compared to the Scott Reefs, which suggests that the distribution of prey items is not a factor [42]. It is also plausible that the observed patterns in the data were driven by differences in the abundance of benthic invertebrates between the two locations. Assessing the abundance of benthic prey was beyond the scope of this study, since invertebrates hide in the interstices of the reef during the day and are difficult to survey accurately. As gonads were not weighed, it is possible that variation in gonad size reflecting reproductive status influenced our results. However, the sex ratios and spawning stages of collected fishes were similar across locations such that additional weight associated with, for instance, egg production is an unlikely factor. Furthermore, the study species are thought to reproduce throughout the year, thus small differences in the timing of sampling would be unlikely to produce disproportionate numbers of reproducing animals between locations. Parasites can also affect condition [75], but no obvious differences in parasite loads were observed between locations when analysing guts.

An alternative explanation for the observed trends is overfishing of teleosts at the Scott Reefs. For instance, it is possible that fishers have removed significant quantities of mesopredatory teleosts at this location, driving competitive release in the remaining piscivores. However, teleost-targeted fishing at the Scott Reefs is thought to be limited in nature, as Indonesian fishers focus predominantly on sharks, sea cucumbers and other high-value items and have limited abilities to transport large quantities of fishes [41]. Teleost fishing at the Scott Reefs is also an unlikely explanation as many of the mesopredatory fish species supposedly targeted by fishers for subsistence are more abundant at the Scott Reefs relative to the Rowley Shoals [42].

Irrespective of their cause, high indices of condition are likely to have major effects on the reproductive success of mesopredatory fishes. Fish in good condition are more fecund, spawn earlier and produce larger eggs that give rise to more viable larvae than those produced by fish in poorer condition [90–92]. Given that genetic and demographic studies show that the fish and coral communities of these oceanic coral reefs are largely self-seeding [93], improved condition of mesopredatory fishes may directly contribute to the greater abundances of these species at the Scott Reefs compared to the Rowley Shoals. This does not require strong stock-recruitment relationships, even though there is evidence for their existence on coral reefs [93], because maternal contributions play a major role in survivorship of new recruits on coral reefs via mortality bottlenecks [94]. If better condition of adults is indeed aiding reproductive output and survivorship of young, as a number of studies suggest is likely, this could provide a means for differences in the abundance of mesopredatory fishes between these isolated reef systems to “snowball” through time.

Variation in the diet of mesopredatory fishes between locations is likely to have important implications for energy flows, community composition and top-down control of invertebrate populations on coral reefs. Although reef sharks may not be apex predators, our results suggest that their loss may still have the capacity to alter the foraging behaviour, diet and fitness of mesopredatory fishes through direct predation, risk effects or competition. As most mesopredatory teleosts have different life histories to reef sharks (e.g. shorter generation times) and smaller gape sizes [95,96], such changes are unlikely to “compensate” for the loss of sharks. Juvenile lutjanids and lethrinids are important predators of newly-recruited reef fishes in coral reef ecosystems and greater abundances of these medium-sized predators are likely to influence rates of survival and foraging behaviour in other young fishes, including herbivores and invertivores [97]. Such displacement of lethal and risk effects within the food web has the potential to influence the integrity of the reef system by altering the quantity and type of resources that are consumed. In addition, dietary shifts in mesopredatory fishes may cause benthic invertebrates to undergo lower rates of mortality at the Scott Reefs relative to the Rowley Shoals, assuming that other fishes do not change patterns of feeding to exploit this resource.

Although natural experiments lack the manipulation and replication that have traditionally been considered the “gold-standard” of the scientific method, there is growing consensus that these are one of the few techniques available to test hypotheses at large, ecologically relevant scales [98]. The results of this study show that mesopredatory fishes on two comparable reef systems have very different diets and condition indices that are consistent with variation in the abundance of reef sharks and associated differences in risk and/or competitive effects. Our findings have important implications for reef systems worldwide, because sharks are being removed by fishing from coral reef systems at an unprecedented rate [18,99]. This analysis suggests that continued exploitation of sharks may lead to significant shifts in the diet, condition and therefore reproductive fitness of mesopredatory fishes in these environments that have the potential to propagate through coral reef food webs. Further research is required to ascertain the relative contribution of competition and predation-induced risk effects to the observed outcome, particularly given that lutjanids do not seem to comprise a significant proportion of the diets of reef sharks [56]. In addition, acoustic/archival tagging [100] and/or behavioural observations would help to clarify whether removal of sharks leads to shifts in habitat use and diet among prey and competitors. However, our results strongly suggest that populations of sharks and other predators in marine systems should be protected in order to maintain the health of the broader ecosystem.

## Supporting information

S1 File**Fig A in S1 File**. Frequency distributions by size class (mm) of *L*. *gibbus*, *L*. *decussatus*, *L*. *kasmira*, *M*. *grandoculis* and *L*. *bohar* at the Scott Reefs (grey) and the Rowley Shoals (black), where the size class represents the upper limit of the values. **Fig B in S1 File**. Plot of the length (mm) of *L*. *bohar* vs percentage difference in weight (green), height (orange) and width (blue) at the Scott Reefs compared to the Rowley Shoals, based on regression relationships for each of the measured parameters. **Fig C in S1 File**. Condition indices (K and K_n_) plotted against Log Length (Log L) on the x-axis for focal species at the Scott Reefs (grey) and the Rowley Shoals (black) for, from top to bottom: *L*. *gibbus*, *L*. *decussatus*, *L*. *kasmira*, *M*. *grandoculis* and *L*. *bohar*, where $K=100\times \frac{W}{L^{3}}$ and $K_{n}=\frac{Wobserved}{W_{Standard}}$. W was defined as total body weight of a fish (g), W_observed_ as the recorded weight of an individual (g) and W_standard_ as the weight (g) predicted by the formula *W*_*standard*_ = *a* × L^*b*^. Species-specific coefficients *a* and *b* were sourced from Fishbase. **Table A in S1 File**. Mean fish abundances per site at each location of 16 mesopredators at the Scott Reefs and the Rowley Shoals, with focal species in bold. The values were calculated using the Long Term Monitoring Program (LTMP) database of the Australian Institute of Marine Science (AIMS; (http://www.aims.gov.au/docs/research/monitoring/monitoring.html). Standard error estimates are presented in parentheses. **Table B in S1 File**. Percentage difference in weight (W), height (H) and width (Wi) of each of five mesopredatory fishes at the Scott Reefs (S) relative to the Rowley Shoals (R), where %ΔW = 100×(W_S_-W_R_)/W_R_. H and Wi were substituted into this equation to calculate differences in these variables. Differences were calculated for the median value of length for each species. All median differences were significant (regression analysis), except for comparisons of the H and Wi of *L*. *gibbus*. Species characterised by significant interactions between location and length are marked with an asterisk; parentheses contain % differences at the minimum and maximum lengths. **Table C in S1 File**. Linear regression of (A) Log Weight (Log W) and Log Length (Log L), (B) Height (H) and L and (C) Width (Wi) and L. Log W = b_0_ + b_1_× Log L + [b_2_×DV] + [b_3_×Log(L)*DV], where a dummy variable (DV = 1 for the Scott Reefs, DV = 0 for the Rowley Shoals) was included to test for a main effect (DV) and the presence of an interaction *DV* × log *L*. In the formula, b_o_ was the coefficient of the intercept, b_1_ was the coefficient of log L, b_2_ was the coefficient of the DV, b_3_ was the coefficient of DV×L, DV was (0,1) for the Rowley Shoals (R) and Scott Reefs (S), respectively, and DV×L was only included if the coefficient was significant. For (b) and (c), H and Wi were substituted for W in the same equation, but with no logarithmic transformations. Standard errors are indicated in parentheses. Asterisks indicate significance (* = *p*<0.05;** = *p*<0.001, and *** = *p*<0.0001).(DOCX)
