# Supplementary material for: Diet and condition of mesopredators on coral reefs in relation to shark abundance
Source: PLoS One. 2017 Apr 19;12(4):e0165113. doi: 10.1371/journal.pone.0165113 (PMC5396851; doi:10.1371/journal.pone.0165113)
Supplement: S1 File — Fig A in S1 File. Frequency distributions by size class (mm) of L. gibbus, L. decussatus, L. kasmira, M. grandoculis and L. bohar at the Scott Reefs (grey) and the Rowley Shoals (black), where the size class represents the upper limit of the values. Fig B in S1 File. Plot of the length (mm) of L. bohar vs percentage difference in weight (green), height (orange) and width (blue) at the Scott Reefs compared to the Rowley Shoals, based on regression relationships for each of the measured parameters. Fig C in S1 File. Condition indices (K and Kn) plotted against Log Length (Log L) on the x-axis for focal species at the Scott Reefs (grey) and the Rowley Shoals (black) for, from top to bottom: L. gibbus, L. decussatus, L. kasmira, M. grandoculis and L. bohar, where K=100×WL3 and Kn=WobservedWStandard. W was defined as total body weight of a fish (g), Wobserved as the recorded weight of an individual (g) and Wstandard as the weight (g) predicted by the formula Wstandard = a × Lb. Species-specific coefficients a and b were sourced from Fishbase. Table A in S1 File. Mean fish abundances per site at each location of 16 mesopredators at the Scott Reefs and the Rowley Shoals, with focal species in bold. The values were calculated using the Long Term Monitoring Program (LTMP) database of the Australian Institute of Marine Science (AIMS; (http://www.aims.gov.au/docs/research/monitoring/monitoring.html). Standard error estimates are presented in parentheses. Table B in S1 File. Percentage difference in weight (W), height (H) and width (Wi) of each of five mesopredatory fishes at the Scott Reefs (S) relative to the Rowley Shoals (R), where %ΔW = 100×(WS-WR)/WR. H and Wi were substituted into this equation to calculate differences in these variables. Differences were calculated for the median value of length for each species. All median differences were significant (regression analysis), except for comparisons of the H and Wi of L. gibbus. Species characterised by significant intera [file pone.0165113.s001.docx]

SUPPLEMENTARY MATERIAL

S1 FILE

# Diet and condition of mesopredators on coral reefs in relation to shark abundance

**Plos One | Article**

**Shanta C. Barley^a,b*^, Mark G. Meekan^b^  and Jessica J. Meeuwig^a^**

**a** School of Animal Biology and the Oceans Institute, University of Western Australia, 35 Stirling Highway, Crawley, WA 6009, Perth

**b** Australian Institute of Marine Science, The Oceans Institute, University of Western Australia, 35 Stirling Highway, Crawley, WA 6009, Perth


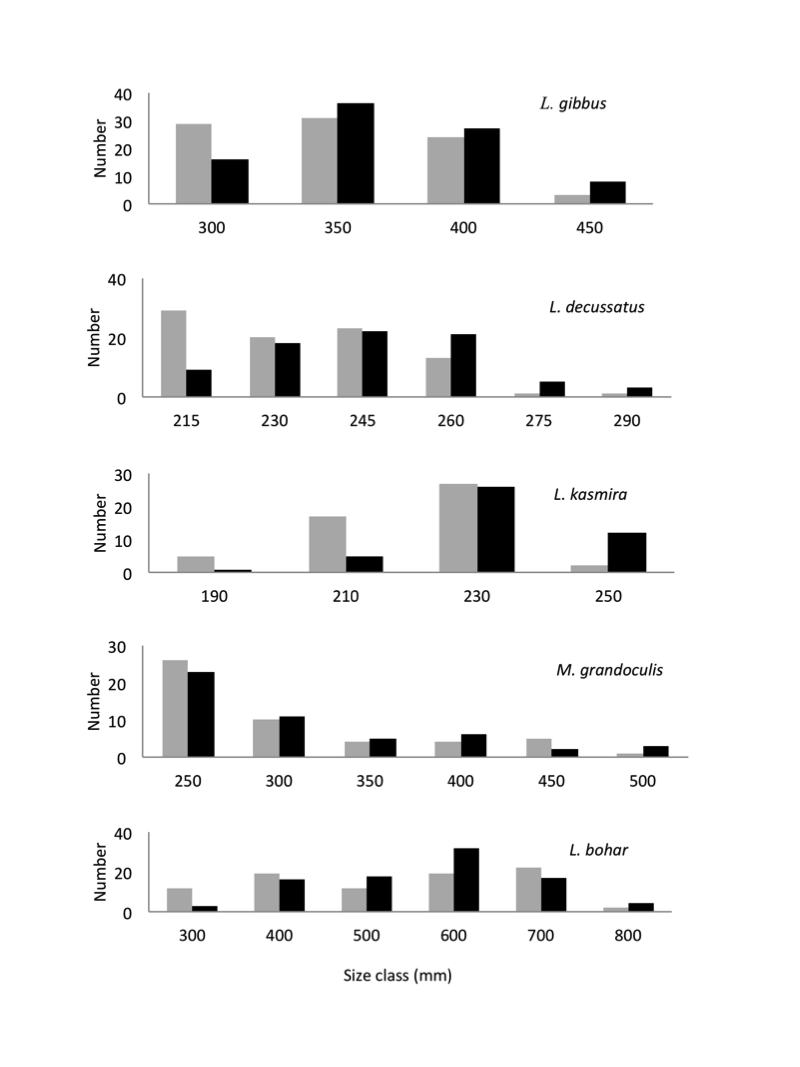


**Figure A in S1 File.** Frequency distributions by size class (mm) of *L. gibbus*, *L. decussatus*, *L. kasmira*, *M. grandoculis* and *L. bohar* at the Scott Reefs (grey) and the Rowley Shoals (black), where the size class represents the upper limit of the values.

**Figure B in S1 File.** Plot of the length (mm) of *L. bohar* vs percentage difference in weight (green), height (orange) and width (blue) at the Scott Reefs compared to the Rowley Shoals, based on regression relationships for each of the measured parameters.

**
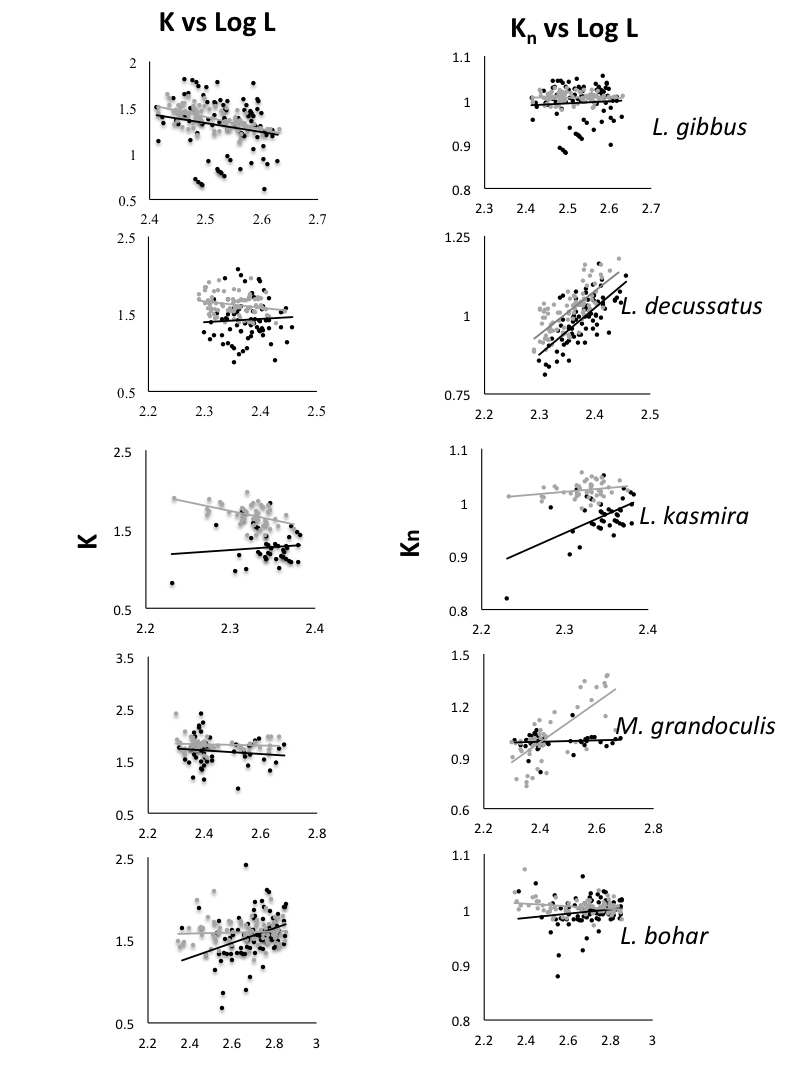
**

**Figure C in S1 File.** Condition indices (K and K_n_) plotted against Log Length (Log L) on the x-axis for focal species at the Scott Reefs (grey) and the Rowley Shoals (black) for, from top to bottom: *L. gibbus, L. decussatus*, *L. kasmira, M. grandoculis* and *L. bohar*, where $K=100\times\frac{W}{L^{3}}$ and $K_{n}=\frac{Wobserved}{W_{Standard}}.$W was defined as total body weight of a fish (g), W_observed_ as the recorded weight of an individual (g) and W_standard_ as the weight (g) predicted by the formula $W_{standard}=a\times L^{b}$. Species-specific coefficients *a* and *b* as defined by [1] were sourced from Fishbase.

**Table A in S1 File.** Mean fish abundances per site at each location of 16 mesopredators at the Scott Reefs and the Rowley Shoals, with focal species in bold. The values were calculated using the Long Term Monitoring Program database of the Australian Institute of Marine Science ([http://www.aims.gov.au/docs/research/monitoring/monitoring.html](http://www.aims.gov.au/docs/research/monitoring/monitoring.html))). Standard error estimates are presented in parentheses.

| Species | Scott Reefs | Rowley Shoals |
| --- | --- | --- |
| ***Monotaxis grandoculis*** | 10.5 (3.6) | 5.8 (0.65) |
| *Epibulus insidiator* | 4.1 (0.52) | 2.9 (1.0) |
| ***Lutjanus decussatus*** | 7.1 (1.3) | 2.5 (1.1) |
| ***Lutjanus gibbus*** | 3.0 (0.71) | 0.88 (0.69) |
| ***Lutjanus kasmira*** | 2.1 (2.1) | 0.83 (0.56) |
| ***Lutjanus bohar*** | 0.38 (0.09) | 0.75 (0.19) |
| *Gnathodentex aureolineatus* | 2.0 (1.3) | 0.57 (0.30) |
| *Macolor spp* | 1.3 (0.43) | 0.40 (0.01) |
| *Cheilinus fasciatus* | 0.52 (0.28) | 0.25 (0.14) |
| *Cheileinus undulatus* | 0.06 (0.03) | 0.24 (0.03) |
| *Lethrinus erythropterus* | 0.91 (0.07) | 0.20 (0.14) |
| *Variola louti* | 0.09 (0.09) | 0.13 (0.07) |
| *Plectropomus areolatus* | 0.2 (0.11) | 0.12 (0.07) |
| *Plectropomus laevis* | 0.12 (0.04) | 0.09 (0.05) |
| *Plectropomus oligacanthus* | 0.96 (0.28) | 0.0 (0.0) |
| *Lutjanus fulvus* | 0.84 (0.84) | 0.0 (0.0) |

**Table B in S1 File.** Percentage difference in weight (W), height (H) and width (Wi) of each of five mesopredatory fishes at the Scott Reefs (S) relative to the Rowley Shoals (R), where %ΔW=100×(W_S_-W_R_)/W_R_. H and Wi were substituted into this equation to calculate differences in these variables. Differences were calculated for the median value of length for each species. All median differences were significant (regression analysis), except for comparisons of the H and Wi of *L. gibbus*. Species characterised by significant interactions between location and length are marked with an asterisk; parentheses contain % differences at the minimum and maximum lengths.

|  | ΔW (%) | ΔH (%) | ΔWi (%) |
| --- | --- | --- | --- |
| *L. gibbus* | 7.6 | ns | ns |
| *L. decussatus* | 14.6 | 4.1 | 5.9 |
| *L. kasmira* | 28.2 | 5.1 | 9.2 |
| *M. grandoculis* | 7.5 | 3.5 | 7.0 |
| *L. bohar* | 8.8 (19.9, -2.4)* | 8.6 | 5.1 (-0.2, 10.3)* |

**Table C in S1 File.** Linear regression of (A) Log Weight (Log W) and Log Length (Log L), (B) Height (H) and L and (C) Width (Wi) and L. Log W = b_0_ + b_1_× Log L + [b_2_×DV] + [b_3_×Log(L)*DV], where a dummy variable (DV = 1 for the Scott Reefs, DV = 0 for the Rowley Shoals) was included to test for a main effect (DV) and the presence of an interaction $DV\times\log L$. In the formula, b_o_ was the coefficient of the intercept, b_1_ was the coefficient of log L, b_2_ was the coefficient of the DV, b_3_ was the coefficient of DV×L, DV was (0,1) for the Rowley Shoals (R) and Scott Reefs (S), respectively, and DV×L was only included if the coefficient was significant. For (b) and (c), H and Wi were substituted for W in the same equation, but with no logarithmic transformations. Standard errors are indicated in parentheses. Asterisks indicate significance (* = *p*<0.05;** = *p*<0.001, and *** = *p*<0.0001).

| **(A) Weight** | nS | nR | df | R^2^ | *p* | MSE | b_0_ | b_1_ | b_2_ | b_3_ | F | adjR^2^ |
| --- | --- | --- | --- | --- | --- | --- | --- | --- | --- | --- | --- | --- |
| *L. gibbus* | 87 | 87 | 173 | 0.75 | 1.4E-51 | 0.08 | -3.98 (0.30)*** | 2.64(0.12)*** | 0.03(0.01)* | ns | 250.9 | 0.74 |
| *L. decussatus* | 87 | 78 | 164 | 0.75 | 1.3E-49 | 0.06 | -4.73 (0.32)*** | 2.95(0.13)*** | 0.06(0.01)*** | ns | 244.0 | 0.75 |
| *L. kasmira* | 49 | 43 | 92 | 0.62 | 1.20E-19 | 0.05 | -3.73 (0.53)*** | 2.51(0.22)*** | 0.11(0.01)*** | ns | 74.0 | 0.62 |
| *M. grandoculis* | 50 | 50 | 99 | 0.96 | 6.3E-68 | 0.06 | -4.63 (0.15)*** | 2.94(0.06)*** | 0.03(0.01)* | ns | 1129.9 | 0.96 |
| *L. bohar* | 86 | 90 | 175 | 0.97 | 9.4E-134 | 0.07 | -3.98 (0.30)*** | 3.30(0.07)*** | 0.49(0.23)* | 0.18(0.09)* | 2017.8 | 0.97 |
| **(B) Height** |  |  |  |  |  |  |  |  |  |  |  |  |
| *L. gibbus* | 37 | 38 | 74 | 0.88 | 1.9E-34 | 4.21 | -8.60 (4.96) | 0.33(0.01)*** | -1.10(0.99) | ns | 275.1 | 0.88 |
| *L. decussatus* | 38 | 35 | 72 | 0.65 | 1.5E-16 | 3.55 | -0.36(5.90) | 0.27(0.03)*** | 2.50(0.91)* | ns | 64.2 | 0.64 |
| *L. kasmira* | 50 | 43 | 92 | 0.6 | 2.2E-18 | 2.38 | 10.6(4.30)* | 0.22(0.02)*** | 2.80(0.54)*** | ns | 66.1 | 0.59 |
| *M. grandoculis* | 50 | 49 | 98 | 0.98 | 5.8E-87 | 2.91 | -1.14(1.22) | 0.31(0.004)*** | 2.96(0.59)*** | ns | 2956.8 | 0.98 |
| *L. bohar* | 39 | 39 | 77 | 0.98 | 3.5E-61 | 5.66 | -2.57(2.80) | 0.28(0.005)*** | 8.10(1.30)*** | ns | 1497.9 | 0.97 |
| **(C) Width** |  |  |  |  |  |  |  |  |  |  |  |  |
| *L. gibbus* | 37 | 38 | 74 | 0.6 | 3.4E-15 | 3.85 | 1.26(4.53) | 0.14(0.01)*** | 1.28(0.90) | ns | 54.9 | 0.59 |
| *L. decussatus* | 38 | 35 | 72 | 0.53 | 2.0E-12 | 2.58 | -4.56(4.30) | 0.16(0.02)*** | 1.82(0.66)* | ns | 40.6 | 0.54 |
| *L. kasmira* | 50 | 43 | 92 | 0.49 | 8.4E-14 | 2.13 | -3.65(3.83) | 0.16(0.02)*** | 2.53(0.48)*** | ns | 42.9 | 0.48 |
| *M. grandoculis* | 50 | 49 | 98 | 0.93 | 3.0E-56 | 2.93 | -1.13(1.23) | 0.14(0.004)*** | 2.74(0.59)*** | ns | 640.7 | 0.93 |
| *L. bohar* | 39 | 39 | 77 | 0.94 | 5.1E-46 | 5.10 | -3.25(3.80) | 0.15(0.007)*** | -5.10(4.80)*** | 0.02(0.01)* | 410.6 | 0.94 |

## Supplementary Methodology

### Other measures of condition

We calculated two indices of condition, including Fulton’s condition factor (K), where K=100×Weight/Length^3^. This is considered to be one of the best measures of condition when body proportions are isometric [2]. In addition to calculating K, we also calculated the relative condition factor K_n_ [3], which is the observed weight of a fish divided by the weight predicted by the equation Weight=a×Length^b^. The resulting values for condition factors were compared between the Scott Reefs and the Rowley Shoals using a paired two-sample t-test. Lastly, we calculated mean residuals for each species using a single regression of Log Weight against Log Length for all individuals observed at both systems [4,5].

Our condition indices confirmed the results of the regression of log-transformed body mass on length. Fulton’s K differed significantly for all species between the Scott Reefs and the Rowley Shoals (paired two-sample, one-tailed t-test, *p=0.*027, n=5), as did K_n_ (paired two-sample, one-tailed t-test, *p=0.*014, n=5). Positive residuals (indicative of fish with greater weight per unit length) were associated with mesopredators from the Scott Reefs, whereas negative residuals were associated with the Rowley Shoals.

# References

1. Froese R. Cube law, condition factor and weight-length relationships: History, meta-analysis and recommendations. Journal of Applied Ichthyology. 2006. pp. 241–253. doi:10.1111/j.1439-0426.2006.00805.x

2. Peig J, Green AJ. New perspectives for estimating body condition from mass/length data: the scaled mass index as an alternative method. Oikos. 2009;118: 1883–1891. doi:10.1111/j.1600-0706.2009.17643.x

3. Le Cren ED. The length-weight relationship and seasonal cycle in gonad weight and condition in the perch (Perca fluviatilis). J Anim Ecol. 1951;20: 201–219. doi:masse poids taille methodologie

4. Reist JD. An empirical evaluation of several univariate methods that adjust for size variation in morphometric data. Can J Zool. 1985;63: 1429–1439. Available: http://www.nrcresearchpress.com.ezproxy.library.uwa.edu.au/doi/pdf/10.1139/z85-213

5. Cone RS. The Need to Reconsider the Use of Condition Indices in Fishery Science. Trans Am Fish Soc. Taylor & Francis; 1989;118: 510–514. doi:10.1577/1548-8659(1989)118<0511:TNTRTU>2.3.CO;2
